# Gene Regulatory Network Inference: Connecting Plant Biology and Mathematical Modeling

**DOI:** 10.3389/fgene.2020.00457

**Published:** 2020-05-25

**Authors:** Lisa Van den Broeck, Max Gordon, Dirk Inzé, Cranos Williams, Rosangela Sozzani

**Affiliations:** ^1^Department of Plant and Microbial Biology, North Carolina State University, Raleigh, NC, United States; ^2^Department of Electrical and Computer Engineering, North Carolina State University, Raleigh, NC, United States; ^3^Department of Plant Biotechnology and Bioinformatics, Ghent University, Ghent, Belgium; ^4^VIB Center for Plant Systems Biology, Ghent, Belgium

**Keywords:** gene regulatory network, network properties, network inference, machine learning, experimental methodologies

## Abstract

Plant responses to environmental and intrinsic signals are tightly controlled by multiple transcription factors (TFs). These TFs and their regulatory connections form gene regulatory networks (GRNs), which provide a blueprint of the transcriptional regulations underlying plant development and environmental responses. This review provides examples of experimental methodologies commonly used to identify regulatory interactions and generate GRNs. Additionally, this review describes network inference techniques that leverage gene expression data to predict regulatory interactions. These computational and experimental methodologies yield complex networks that can identify new regulatory interactions, driving novel hypotheses. Biological properties that contribute to the complexity of GRNs are also described in this review. These include network topology, network size, transient binding of TFs to DNA, and competition between multiple upstream regulators. Finally, this review highlights the potential of machine learning approaches to leverage gene expression data to predict phenotypic outputs.

## From Genes to Networks: A Continuous Molecular Scale for Plant Research

Plant responses need to integrate environmental signals, including those from biotic and abiotic stresses. Additionally, plants integrate intrinsic signals, such as developmental or hormonal cues. Plant responses to environmental and intrinsic signals are under tight control to ensure a fast and appropriate response and at the same time prevent an indiscriminate activation of this response (Swift and Coruzzi, 2017). Accordingly, the chance of randomly activating a plant response is significantly reduced when multiple transcription factors (TFs) regulate and fine-tune this response (Swift and Coruzzi, 2017). As such, multiple upstream TFs, connected to each other, form complex gene regulatory networks (GRNs) to redundantly control downstream responsive genes, also defined as target genes (Hernando et al., 2017). These GRNs consist of nodes that represent genes, and edges that represent the regulatory connections between genes. Overall, GRNs provide a blueprint of the molecular interactions underlying plant responses. The generation of GRNs in the context of plant responses has played a critical role in identifying new regulatory connections between genes and driving novel hypotheses. For example, the generation of a GRN at the base of the myo-inositol metabolic pathway in soybean (*Glycine max*) predicted new regulatory interactions, of which 13 interactions could be validated. The GRN was generated with transcriptome data from two mutant lines, *mips1* (*myo-inositol phosphate synthase 1*) and a triple mutant *mips1/mrp-l (multi-drug resistance protein)/mrp-n* that led to low phytic acid and a decrease in seed emergence (Redekar et al., 2017). More specifically, differentially expressed genes (DEGs) were clustered in modules based on their expression patterns. Putative regulatory interactions between the DEGs encoding TFs and the different modules were then determined based on the enrichment of known DNA-binding motifs within each module (Redekar et al., 2017). By using a systems-level approach, unknown regulatory interactions were predicted and validated, allowing for a better understanding of the myo-inositol metabolic pathway in soybean.

In another example, newly identified hub genes, i.e., highly connected genes, were hypothesized to have functional roles as stress-induced genes (Vermeirssen et al., 2014). To generate the stress-induced GRN, an *Arabidopsis* microarray compendium including 199 abiotic stress conditions was used to identify modules of co-expressed genes. Using three different network inference techniques, a set of putative upstream TFs was identified for each module resulting in a total of 200,014 regulatory interactions. Fifty percent of the predicted regulatory interactions involving seven identified hub TFs were confirmed, highlighting the capacity of GRNs to identify functional interactions (Vermeirssen et al., 2014). Furthermore, one of these seven TFs, NAC DOMAIN CONTAINING PROTEIN 32 (NAC032), was not yet shown to play a role in stress tolerance. Phenotypic analyses confirmed the involvement of NAC032 in the regulation of the osmotic stress response, demonstrating the power of GRNs to identify regulatory TFs in a biological context (Vermeirssen et al., 2014).

In addition to identifying new regulatory connections between genes with GRNs, the assessment of GRN topology can provide a system-level approach to understand network complexity and robustness, and help in identifying putative strategies for manipulating the network response. The network topology refers to the structure of the GRN and includes properties such as node connectivity, network diameter, network density, and network motifs (Hu et al., 2005). Node connectivity is the number of connections a node has to other nodes. Network diameter measures the number of connections between the most distant parts of the network. Network density is a measure of the number of connections in a network in proportion to the number of nodes. Lastly, network motifs are subgraphs that occur within a GRN with high occurrence. These aspects of network topology contribute to the understanding of network robustness and complexity.

## Biological Properties of Gene Regulatory Networks and Approaches to Investigate Them

**FIGURE 1:**
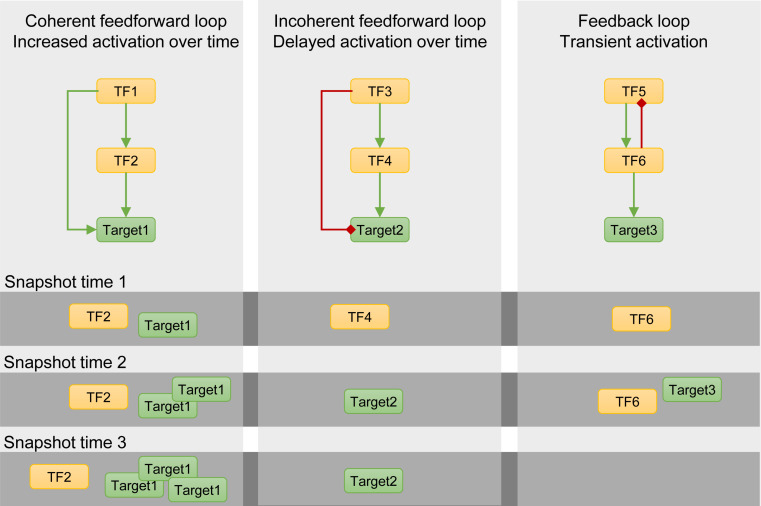
Schematic representation of multiple snapshots of the transcriptomes in relation to the presence of network motifs, such as feedforward and feedback loops. **Left panel:** a coherent feedforward loop composed of activation interactions results in increased activation of the target gene over time as the induction of the second transcription factor (TF) only occurs after its own activation by TF1. **Middle panel:** delayed activation of target2 as a result of the delayed activation of TF4, part of an incoherent feedforward loop. **Right panel:** as a result of the feedback loop between TF5 and TF6, target3 is only transiently activated. These interactions also depend on the relationship between the two TFs, the degradation of the transcripts, and the amount of input signal. The observed transcriptomes will thus be different over multiple time points and result in different snapshots (dark gray zones). Green and red arrows represent activation and repression, respectively.

As mentioned above, complex GRNs can be identified that contribute to plant development and environmental responses. Several biological properties, including network topology, contribute to the complexity of GRNs and can be assessed when studying GRNs:

1. *Multiple upstream regulators*: Many genes are regulated by multiple upstream TFs, resulting in a complex regulatory module for every gene (Barah et al., 2016; Huang et al., 2017). Moreover, upstream TFs can act alone, form complexes, compete for binding, and act as a co-factor with or sequester other TFs (Nagel and Kay, 2012). In addition to the high number of upstream regulators, some TFs only regulate a downstream gene in combination with another TF and/or under specific conditions (Gonzalez et al., 2015). Such interactions are thus overlooked in the absence of the second TF. Furthermore, it has been shown that TFs bind to different motifs when paired with other TFs than motifs bound by single TFs, further increasing network complexity (Jolma et al., 2015). How multiple upstream TFs regulate the expression of one target gene is thus highly complex. Currently, transient luciferase assays (TEAs) can be used to quantify the effect of multiple TFs on the expression of a target gene (Vanden Bossche et al., 2013). Accordingly, by transforming protoplasts with multiple effector plasmids containing the TFs of interest and one reporter plasmid with the promoter of the target gene of interest, the combined effect of these TFs on the activity of the promoter can be evaluated. This information can be used to refine the network.
2. *Transient binding*: Transcription factors scan the DNA until they encounter the correct DNA-binding motif and bind to the DNA, which can occur transiently. A TF can execute its function through the hit-and-run principle, which means that once the TF is bound (*hit*), it establishes a transcriptional complex that regulates transcription even when the TF is no longer present (*run*) (Doidy et al., 2016; Swift and Coruzzi, 2017). Because these transient bindings occur within minutes and do not last, they are harder to detect by methods such as chromatin immunoprecipitation (ChIP), resulting in false negatives in the GRN. Performing ChIP experiments with an inducible system over multiple time points can decrease the number of false negatives (Doidy et al., 2016; Swift and Coruzzi, 2017). As such, a new class of target genes that is only transiently bound by basic LEUCINE ZIPPER 1 (bZIP1) within 1 to 5 min and not at later time points was discovered (Para et al., 2014).
3. *Size*: Depending on the molecular process, the network size can increase significantly, reaching hundreds of genes in one network. Researchers can reduce the number of genes in the network by (i) increasing the fold change or decreasing the *q*-value threshold to select a smaller subset of DEGs, (ii) focusing on a specific type of protein such as TFs, or (iii) performing an overlap with DEGs from other relevant datasets. To visualize, explore, and analyze these networks, regulatory interactions can be uploaded in Cytoscape^®^ and analyzed with different applications such as BiNGO or NetMatch^∗^ (Su et al., 2014). Generally, these large-scale networks include hub genes with a high out-degree, i.e., the number of outgoing edges and thus the number of target genes (Lorenz et al., 2011; Barah et al., 2016). Such hub genes can be biologically important genes and thus relevant for further studies characterizing gene function.
4. *Network topology*: Within a GRN, multiple network motifs, such as feedback and feedforward loops, are found (Nohales and Kay, 2016). These network motifs can exhibit specific dynamic characteristics (Figure 1). Depending on the network motif, delayed, transient, or increased activation of target genes can occur (Figure 1; Martin et al., 2016). Thus, as a result of their dynamic behavior, network motifs contribute to GRN dynamics and complexity (Figure 1). As shown in Figure 1, multiple snapshots of the transcriptomes can be detected depending on the sampled time point (Figure 1). These characteristics were highlighted in Chang et al., where ChIP-seq data identifying EIN3 targets upon ethylene treatment were combined with RNA-seq analysis to construct a GRN (Chang et al., 2013). Because samples were taken at multiple time points after ethylene treatment, the dynamics of the response to ethylene could be unraveled. This study shows the power of time courses to unravel the dynamics of a GRN and view the progression of the downstream events (Chang et al., 2013).

The latter network topology also contributes to the phenotypic output of plant responses. For example, incoherent feedforward loops will generate pulses of gene expression, which in turn generate rhythmic behaviors, such as the circadian clock in *Arabidopsis* (Joanito et al., 2018). Studying phenotypic outputs is commonly achieved by eliminating or overexpressing a single gene or several genes. However, studying phenotypic outputs in the context of entire GRNs appears to be more challenging, and additional tools may be necessary to connect network characteristics and plant phenotype.

## Experimental Methodologies to Generate Gene Regulatory Networks

**FIGURE 2:**
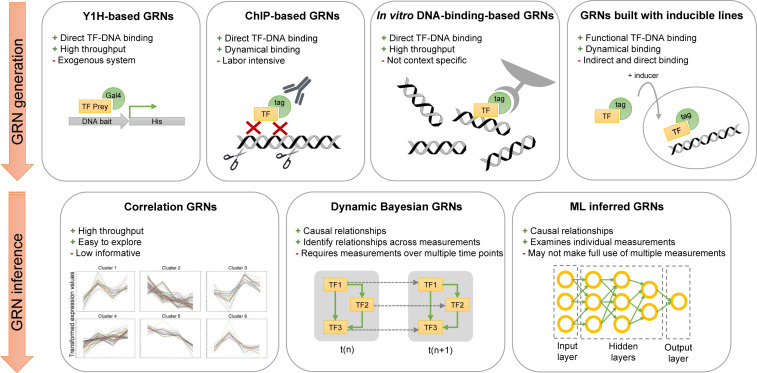
Overview of network generation and inference methodologies described in this review. High-throughput Y1H screens, ChIP-seq assays, *in vitro* DNA-binding experiments, or expression experiments with inducible overexpressing lines can be used to generate GRNs. Three computational methodologies are described in this review to infer GRNs: Correlation networks, Dynamic Bayesian networks, and machine learning networks. Advantages and disadvantages are given for each experimental and computational methodology. GRN, gene regulatory network; ML, machine learning; Y1H, yeast one-hybrid; ChIP, chromatin immunoprecipitation.

To reach a comprehensive understanding of plant responses, multi-level data, ranging from phenotypic analyses to gene expression analyses, are being acquired. Advances in bioinformatics and high-throughput experimental approaches, such as RNA sequencing and ChIP sequencing, allow us to study whole transcriptomes. This variety of data can be used to study genes across a molecular scale, ranging from a single gene, several genes, or interacting genes forming a GRN. A variety of experimental methodologies are used to collect data for the generation of GRNs and provide a system-level view of the plant response under study (Figure 2). These methodologies can (i) determine the binding of a TF to specific DNA sequences or (ii) identify target genes that are regulated by a TF of interest. Based on this information, directional edges can be drawn from the genes encoding TFs to their downstream targets.

Methodologies to identify DNA binding events of TFs are yeast one-hybrid (Y1H) assays, ChIP experiments and *in vitro* DNA binding assays (Figure 2). These methodologies are frequently used in studies focusing on the detailed characterization of a single gene or a small group of genes. Additionally, they can be applied in a systems-level context when performed in parallel.

– **Y1H Screens.** A large-scale Y1H screen that tested the promoters of 50 genes involved in xylem development against 467 TFs was used to construct a GRN at the base of secondary cell wall synthesis (Taylor-Teeples et al., 2015). This Y1H screen resulted in a highly interconnected GRN containing feedforward loops and led to the identification of new key TFs in the specification of the secondary cell wall (Taylor-Teeples et al., 2015). Another recently published GRN constructed from Y1H screens unraveled a GRN downstream of plant cell regeneration; subdivided this GRN in wounding, auxin, or cytokine-induced regeneration subnetworks; and identified hub TFs and novel promoter–TF interactions (Ikeuchi et al., 2018). Even though Y1H assays allow for high-throughput data generation of direct TF-DNA binding to construct GRNs, the yeast genetic background can affect the results and the identified regulatory interactions should be confirmed *in planta*.
– **ChIP.** When performing ChIP followed by high-throughput sequencing (ChIP-seq) or microarray hybridization (ChIP-chip), genome-wide TF binding loci can be determined. Although ChIP-seq is limited to one TF, the technique can be used to build GRNs when performed in parallel. A recently published study performed ChIP-seq experiments on 21 TFs related to abscisic acid (ABA) in the presence and absence of ABA, enabling the identification of dynamic TF binding; for 19 of the 21 TFs, the binding events increased after ABA treatment (Song et al., 2016). Because the authors determined the direct downstream targets of 21 TFs, they could identify highly regulated target genes that were downstream of multiple TFs, such as core ABA genes but also novel non-ABA-related genes, such as *RGL3* (*RGA-like 3*) regulated by gibberellin (GA) and *ACS2* (*ACC synthase 2*) controlling the biosynthesis of ethylene (Song et al., 2016). Expresso is available to explore and access available processed ChIP-seq data in *Arabidopsis* (Aghamirzaie et al., 2017).
– ***In vitro* DNA-Binding Experiments.** As with Y1H assays, this methodology can be used to construct GRNs; however, the large number of regulatory interactions found with these techniques are not always functional and need to be placed in a biological context. *In vitro* techniques used to determine DNA binding events of TFs include protein binding microarrays (PBM), DNA-affinity purification sequencing (DAP-seq), and Systematic Evolution of Ligands by Exponential Enrichment (SELEX). PBMs consist of dsDNA microarrays that are incubated with a tagged TF of interest. The DNA-bound TFs are detected with a fluorescent-bound antibody (Berger and Bulyk, 2009). Using PBMs, the DNA-binding motif of 2913 TFs, selected from different species, was determined in a large-scale experiment (Weirauch et al., 2014). These data are publicly available at Cis-BP^1^ and forms a large resource for bioinformatics analysis and GRN inference. DAP-seq and SELEX are similar techniques; however, to our knowledge SELEX has not been used to build a GRN in plants. For SELEX, a target (e.g., TF) is incubated with a library, e.g., a synthetic library or a genome-based library of ssDNA, dsDNA, or RNA, followed by the selection and amplification of the bound complexes (Djordjevic, 2007). DAPseq makes use of a dsDNA library (inferred from genomic DNA) of which the fragments contain an adaptor sequence. A purified TF bound to beads is added to the library. Next, the bound gDNA fragments are eluted and sequenced. By mapping the sequence reads onto the genome, bound target genes can be identified (Bartlett et al., 2017). The *in vitro* DNA-binding sites of 526 *Arabidopsis* TFs are determined with DAP-seq (O’Malley et al., 2016)^2^.

In addition to constructing a GRN based on the binding events of a TF, gene expression data of inducible overexpressing plant lines can be used to build GRNs (Figure 2). The major advantage of inducible overexpressing lines is that the desired gain or loss of function can be applied at a specific developmental stage, resulting in temporal or developmental specific GRN changes. Three inducible systems are generally used. (i) TFs translationally fused to a glucocorticoid receptor (GR) domain translocate to the nucleus in the presence of dexamethasone (DEX) (Corrado and Karali, 2009). The two other systems make use of a two-component system in which a chimeric TF induces the expression of the transgene upon a chemical inducer. (ii) First, a fusion protein, called XVE, contains a LexA DNA binding domain, the VP16 transactivation domain, and the human estrogen receptor domain and is activated when treated with estrogen (e.g., estradiol). Subsequently, the fusion protein can activate the expression of the TF of interest by binding on the LexA operator sequence upstream of the gene encoding the TF (Zuo et al., 2000). (iii) The third system, called the *alc* system, also contains two components: the first component is the AlcR TF activated in the presence of ethanol or acetaldehyde and the second component consists of the gene encoding the TF of interest downstream of the AlcA promoter. When the AlcR is active, it can bind the AlcA promoter and induces the expression of the TF of interest (Caddick et al., 1998).

These systems have been used to overexpress a gene of interest at a desired developmental stage and explore their downstream effects with, e.g., transcriptomics (Wellmer et al., 2006; Dubois et al., 2013). For example, *APETALA1* (*AP1*), a central gene in the initiation of flower development, was fused to a GR-domain and transformed into the *ap1 cal* (*cauliflower*) double mutant. By specifically activating *AP1* in the inflorescence meristems of this mutant, the temporary obstruction of flower formation in *ap1 cal* is lifted and flowers develop synchronously (Wellmer et al., 2006). In addition to inducing TFs, a system has been developed in which artificial microRNAs (amiRNAs) are specifically induced during flower development, generating new possibilities to unravel GRNs (O’Maoileidigh et al., 2015).

These GRNs contain experimentally determined transcriptional regulations but do not make a distinction between indirect or direct targets. By using cycloheximide in combination with inducible overexpressing lines, indirect and direct target genes can be distinguished. Cycloheximide will block the formation of new proteins, preventing direct targets to in turn regulate their targets and thus the detection of indirect target genes (Davies and Exworth, 1973). Based on these principles, the technique TARGET (Transient Assay Reporting Genome-wide Effects of Transcription factors) was developed (Bargmann et al., 2013). Protoplasts are transformed with a GR-TF fusion cassette that also contains a red fluorescent protein (RFP), enabling the sorting of transformed protoplast through fluorescence-activated cell sorting (FACS). With the addition of 4-thiouracil (4tU), a distinction can be made between existing and newly synthesized mRNA (Doidy et al., 2016). Using this technique, the “hit-and-run” principle was proven for bZIP1 (Para et al., 2014). However, some genes are transcriptionally induced by cycloheximide, which can render false positive. In this case, including early and later time points upon induction of overexpression can indicate whether DEGs are direct or indirect downstream targets (Van den Broeck et al., 2017). As such, the regulatory effect of 21 TFs on their downstream targets was assessed upon multiple time points after induction of overexpression. Genes differentially expressed 1, 2, and 4 h after overexpression were selected as putative direct targets and experimentally validated. The validated targets were used to construct a GRN that is specifically activated upon osmotic stress (Van den Broeck et al., 2017).

The above-described methodologies use experimental data ranging from Y1H screens to expression data, to construct GRNs. However, these methodologies introduce uncertainties as a result of incomplete observations, background noise, and systematic errors, leading to false negatives. To this end, researchers can make use of network inference approaches to describe regulatory interactions as probabilities and built GRNs.

## Probabilistic Network Inference Approaches to Identify Causal Relations

The inference of GRNs from large datasets is not an easy task, and different computational tools, including correlation networks, and causal inference methods such as Mutual Information and Bayesian networks, have been applied to this task (Margolin et al., 2006; Vignes et al., 2011). Co-expressed genes can be identified from microarray or RNAseq data with correlation methods, such as Pearson or Spearman correlation. This information can then be used to build correlation networks (Figure 2). These correlation networks are based on the principle that genes expressed in the same conditions could perform a similar biological function. Correlation networks can thus be powerful tools to predict new regulatory genes of a specific plant response. For example, a correlation network in rice was built based on 57 microarray experiments performed during different stages of anther development. This resulted in 545 clusters, with genes showing the same expression pattern across the different samples (Lin et al., 2017). By mapping DEGs identified with knock-out experiments onto the correlation network, new biologically important genes involved in anther development were identified. GRNs have been developed for a large number of species under different environmental conditions and multiple tools are available to explore correlation networks or identify sets of co-expressed genes (Table 1; De Bodt et al., 2010).

**TABLE 1:** Summary of the available tools to explore expression datasets in different species.

Tool	Species	Specificity	References
CORNET	*Arabidopsis thaliana*	Co-expression and protein-protein interaction tool	De Bodt et al., 2010
FlowerNet	*Arabidopsis thaliana*	Includes only stamen-, pollen-, or flower-specific expression studies	Pearce et al., 2015
Genevestigator	*Arabidopsis thaliana, Hordeum vulgare, Oryza sativa, Medicago truncatula, Glycine max, Zea mays, Nicotiana tabacum, Solanum lycopersicum, Physcomitrella patens, Triticum aestivum*, and *Sorghum bicolor*	Multiple tools to analyze a set of genes, such as clustering and differential expression	Hruz et al., 2008
RapaNet	*Brassica rapa*	Includes 143 B. rapa microarrays	Kim et al., 2017
RiceAntherNet	*Oryza sativa*	Includes 57 rice anther tissue microarrays	Lin et al., 2017
RiceArrayNet/PlantArrayNet	*Oryza sativa, Arabidopsis thaliana, and Brassica rapa*	Includes diverse microarrays and links genes to pathway maps	Lee et al., 2009
PlantExpress	*Oryza sativa and Arabidopsis thaliana*	Contains two sub platforms, OryzoExpress and ArthaExpress, enabling cross-species analysis	Kudo et al., 2017
ATTED-II	*Arabidopsis thaliana, Brassica rapa, Oryza sativa, Glycine max, Populus trichocarpa, Solanum lycopersicum, Vitis vinifera, Medicago truncatula*, and *Zea mays*	Includes microarray data of crops and added RNAseq data of *Arabidopsis*	Obayashi et al., 2014, 2018
PlaNet	*Arabidopsis thaliana, Hordeum vulgare, Medicago truncatula, Populus trichocarpa, Oryza sativa, Glycine max, Triticum aestivum, Nicotiana tabacum, Brachypodium distachyon, Physcomitrella patens, and Selaginella moellendorffii*	Comparative analysis of co-expression networks across plant species and prediction of gene function	Mutwil et al., 2011
PLANEX	*Arabidopsis thaliana, Glycine max, Hordeum vulgare, Oryza sativa, Solanum lycopersicum, Triticum aestivum, Vitis vinifera, and Zea mays*	Contains microarray data from the Gene Expression Omnibus (GEO)	Yim et al., 2013

*Different tools are developed to identify sets of co-expressed genes across a wide range of environmental conditions or mutant lines and explore these regulatory modules. Each tool has overlapping and distinct features.*

Correlation networks can be used to explore large datasets and identify putative central regulators/hub genes (Figure 2). However, these networks are unable to provide information about transcriptional relations between upstream regulators and downstream target genes. They are also limited in determining whether the interaction is direct or indirect, results in activation or repression, or involves competition between multiple upstream regulators. One technique to provide useful predictions using correlation networks despite this limitation is to integrate additional types of data. For example, combining correlation networks with metabolic data has led to the identification of key regulatory genes in metabolic pathways (Wu et al., 2016). The addition of genome-wide association studies (GWAS) can increase the power and robustness of a correlation network. A correlation network at the base of mild and severe salt stress response in roots was constructed in parallel with a GWAS of a 94-RIL (Ler/Cvi) population. Genes identified with GWAS were used to explore the clusters of the correlation network. By analyzing the neighboring genes of the identified GWAS hits, connections could be made, such as the allocation of GWAS and neighboring genes identified under mild salt stress to specific clusters (Kobayashi et al., 2016). Leveraging the advantage of combining GWAS with correlation networks, a computational framework, Camoco, was built to identify candidate SNP-associated genes, build a correlation network, and prioritize the candidates genes based on their expression correlation (Schaefer et al., 2018). This approach is especially useful for species for which the majority of the genome remains functionally uncharacterized. Other methods that integrate correlation networks with additional data are based on known DNA-binding motifs to identify the upstream regulators of a group of DEGs that cluster together (Palaniswamy et al., 2006; Lv et al., 2014; Barah et al., 2016). The TF2Network tool is such a method that allows constructing a GRN based on DNA-binding motifs by searching in a given list of genes for enriched TF-binding sites (Kulkarni et al., 2017).

While correlation networks are an adaptable and widely used computational tool, other methods are necessary to infer causal relationships from gene expression without the use of DNA-binding motifs. Using network inference methods, putative upstream regulators for DEGs can be predicted by searching for regulators that can explain observed gene expression patterns, allowing the researcher to construct a GRN (Segal et al., 2003; Phuong et al., 2004). Bayesian network (BN) inference provides one avenue to construct large, informative GRNs and infer direct causal relations between genes (Figure 2; Yu et al., 2004; Chen et al., 2006; Bansal et al., 2007; Vignes et al., 2011). In BNs, edges are encoded as probabilistic connections between their origin and destination nodes (Pearl, 2008). These networks are a particularly widely used tool in determining conditional dependencies among genes to predict direct interactions between an upstream gene and its downstream targets (Yu et al., 2004; Chen et al., 2006; Bansal et al., 2007; Vignes et al., 2011). In one example, a BN was used to infer conditional dependencies among *SHOOT MERISTEMLESS* (*STM*) and 56 other genes encoding TFs with publicly available datasets in *Arabidopsis*. With this network a strong dependency was identified between *STM* and *CUP-SHAPED COTYLEDON 1* (*CUC1*), which was then experimentally validated (Scofield et al., 2018). Importantly, BNs can be constructed by beginning with a set of genes of interest and iteratively adding genes that lead to a model with increased fitness. Using this approach, several *GATA* TFs were identified as possible regulators of photosynthesis in *Arabidopsis* and novel relationships were tested (Needham et al., 2009).

To lower the number of possible networks and thus sometimes extensive computation time, network inference based on Bayesian principles can make use of *a priori* knowledge about the pathway. *A priori* knowledge can be incorporated in ways such as restricting possible network structures based on known patterns of interaction or limiting the number of connections any node may have. For example, Bayesian inference with an assumption of hierarchical structure and a limited number of connections was applied to infer GRNs in *Arabidopsis* under different stress conditions. These networks identified 9 TFs as putative regulators of *DESICCATION-RESPONSIVE PROTEIN 29A* (*RD29A*), a well-known stress-induced gene, in agreement with previous experimental data (Penfold et al., 2012).

Another method to infer regulatory relationships is the use of ordinary differential equation (ODE) models. These approaches are based on fitting parameterized differential equations to time-course expression data, where these equations characterize the dynamic influence of regulators on the expression patterns of target genes. These equations typically describe mechanistic interactions between regulators and targets and can vary in complexity, ranging from linear equations to more complex non-linear representations (Wu et al., 2014). Given a specific model type and time-course gene expression data, optimization routines are used to estimate the parameters of the ODE. These include least-squares methods, LASSO, Markov Chain Monte Carlo, and Genetic Algorithms (Locke et al., 2005, 2006; Krouk et al., 2010; Koryachko et al., 2019). Issues that arise when using ODEs to model GRNs include overly complex models resulting in overparameterization, sparse data resulting in unidentifiable parameters (Krouk et al., 2010), overfitted parameters resulting in models that are not generalizable (Krumsiek et al., 2010), and model structures that result in “sloppy” parameters where a wide range of parameters provide adequate fit to the data (Bujdoso and Davis, 2013). ODE models are also typically constrained to a subset of DEGs to reduce the numbers of parameters that need to be optimized. Putative upstream regulators of genes involved in the response to different light conditions in *Arabidopsis* were selected based on literature, databases such as Kyoto Encyclopedia of Genes and Genomes (KEGG), and regulator-gene predictions based on motif presence in promoter regions. Fitting ODE models to time-course expression data allowed for the removal of weak regulatory interactions and the refinement of a GRN under photosynthetic light acclimation (Yao et al., 2011). Similarly, an ODE model incorporating hidden states to represent actual protein abundances was used to infer GRNs related to nitrate response in *Arabidopsis*. In this study, SPL9 was identified as a possible regulator of nitrate signaling and experimentally validated by overexpressing *SPL9* (Krouk et al., 2010).

Importantly, each inference technique has specific advantages and limitations. For example, Bayesian inference methods are well-suited to extract useful information from noisy gene expression data and to identify linear cascades (Marbach et al., 2012). However, they cannot scale to infer large networks and are limited in identifying feedforward loops (Marbach et al., 2012). These shortcomings can be addressed by performing a clustering step prior to inference (de Luis Balaguer et al., 2017) and extending the BN into a Dynamic Bayesian Network (DBN), respectively (Friedman et al., 1998). In DBN inference, a time-course dataset is provided to predict probabilistic dependencies between genes. As such, the value of each gene at one time point depends on the values of its regulators at the previous time point and/or at the same time point, depending on the sparsity of the time-course data that is provided. DBNs have been used to predict mechanisms that are key in regulating circadian rhythms in *Arabidopsis*. These were later confirmed in experimentally verified networks (Dondelinger et al., 2012). Moreover, DBNs have successfully been used to infer GRNs underlying molecular responses and reconstruct experimentally determined stem cell networks. Accordingly, a DBN inferred from root stem cell-specific time-course data identified *PERIANTHIA* (*PAN*) as an upstream of known stem cell regulators. Experimental evidence showed that this newly predicted stem cell regulator indeed controls columella stem-cell maintenance and QC division (de Luis Balaguer et al., 2017). Importantly, the computational pipeline used in this work, called GENIST, was made available on GitHub and through TuxNet, a simple graphical user interface for processing of RNAseq data and inferring GRNs (de Luis Balaguer et al., 2017; Spurney et al., 2019). In addition to TuxNet, other tools are available to facilitate the use of BNs and DBNs for plant biologists, such as BNArray, a tool developed in R that creates small DBNs and combines them to predict regulatory subnetworks (Chen et al., 2006). Similarly, open source Cytoscape plugins are available for network inference: (i) NetworkBMA uses Bayesian Network Averaging to infer regulatory networks (Fraley et al., 2014); (ii) Cygenexpi is based on ODEs and uses known putative regulations and time-course data to assess regulatory interactions (Modrák and Vohradskı, 2018); and (iii) ARACNE can analyze and integrate high-throughput expression steady-state data and was already successfully used in identifying previously known and new transcriptional regulations in the *Arabidopsis* root (Margolin et al., 2006; Chávez Montes et al., 2014).

## Bridging the Gap Between Quantitative Expression Data and Phenotypic Traits With Machine Learning Approaches

**FIGURE 3:**
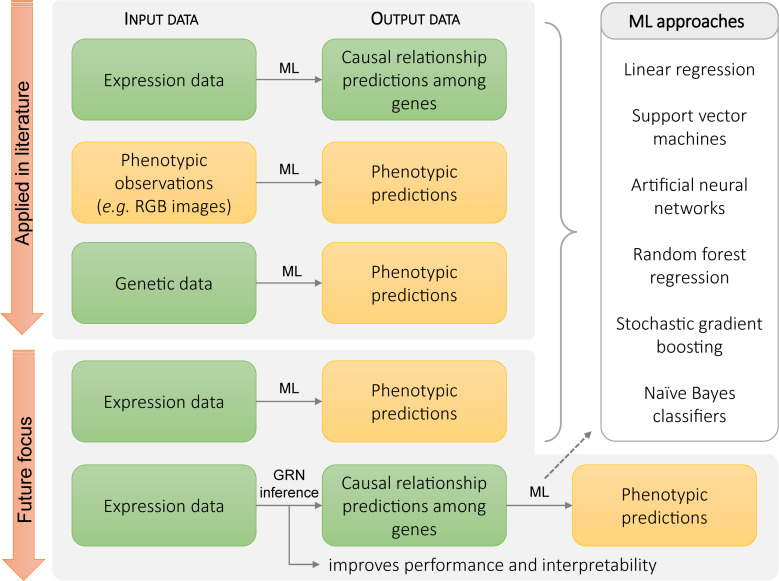
Current and potential future applications of machine learning methods in plant biology. **Top panel**: current applications of machine learning approaches include predicting relationships from expression data, predicting phenotype from direct observational data, and predicting phenotype from genotype. **Bottom panel**: in the future, gene expression data and GRN inference methods could be used to make phenotypic predictions based on the regulatory relationships between genes.

Pleiotropic effects can be a major challenge in making targeted changes to biological systems. This problem can be circumvented by adjusting the specificity of the downregulation or upregulation of the gene expression. For example, the adverse effect of the constitutive overexpression of *PLASTOCHRON1* (*ZmPLA1*) in maize, such as the absence of flowering, is eliminated by targeting the ectopic expression of *PLASTOCHRON1* (*ZmPLA1*) to the transition zone of a maize leaf. This is achieved by placing *ZmPLA1* downstream of the *GA2-OXIDASE* (*ZmGA2OX*) promotor, of which the expression is limited to the transition from cell division to cell expansion and results in larger leaves (Sun et al., 2017). Predicting the need for these kinds of targeted interventions requires a detailed understanding of the complex connections between gene expression data and downstream phenotypic effects. Unraveling GRNs and understanding their dynamics provides one means to link gene expression and phenotype. However, when the link between gene expression and phenotypic output is unclear, unresolved, or highly complex machine learning (ML) approaches can provide an attractive avenue. ML approaches can yield data-driven models that offer predictions, thus providing a broadly applicable toolset to analyze biological data and predict phenotypic outputs based on gene expression data (Figure 3). This could help to improve the effectiveness and precision possible in modifying phenotypic traits.

Machine learning tools have been applied to biological systems at multiple scales. They have been applied to gene expression data to identify DEGs (Pirooznia et al., 2008) and transcriptional regulations between genes (Figure 2; Huynh-Thu et al., 2010). At the phenotypic level, ML systems have been used to analyze images for rapid phenotyping (Gonzalez-Sanchez et al., 2014; Sommer et al., 2017). Computer vision systems using ML have been used to track *Arabidopsis* growth and movement through day–night cycles, extracting patterns of movement and growth, automating extraction of phenotypic information (Bernotas et al., 2019). In another example, linear regression, support vector machines (SVMs), artificial neural networks (ANNs), random forest regression, and stochastic gradient boosting were tested for accuracy and robustness in yield prediction in almonds using orchard images, orchard-specific attributes, and weather data. After testing these ML methods, stochastic gradient boosting was found to provide the best performance in yield prediction and identifying key determinants of almond yield, such as orchard age and levels of precipitation during periods of pollinator activity (Zhang et al., 2019).

Additionally, several ML approaches such as SVMs, random forests, logistic regression, naïve Bayes classifiers, and ANNs have already been applied to genetic data for the prediction of phenotypic traits (Figure 3). For example, deep ANNs were used to predict yield in maize from genotype data and weather conditions. In this case, the models were able to predict yield with a root mean squared error of 12%, although this was highly sensitive to weather prediction accuracy (Khaki and Wang, 2019). ML approaches have also been used to predict genotypes. Logistic regression and naïve Bayes approaches have been used to predict the genotype of crosses between maize strains, with prediction accuracy between 82 and 85% (Seka et al., 2019). However, because of the complexity of ML approaches and lack of interpretable intermediary results, it can be difficult to understand whether the model will generalize well and operate on a wide range of input data without prohibitive amounts of testing. One approach to address this is to identify informative features that can be extracted from the data before it is used in the ML system. Extracting information about this process and using that as an input to the ML system can reduce the complexity of the relationships the ML system needs to infer.

Gene regulation is an integral mechanism for numerous biological processes. As a result, GRN topology plays a significant role in the plant response to intrinsic or environmental signals (Stelling et al., 2002). This connection between phenotype and regulatory relationships makes constructed or inferred GRNs an attractive intermediary step between expression-level data and phenotypic predictions. Due to the key role of gene regulation in determining phenotype, features derived from the topology of GRNs, such as node connectivity, network diameter, and network density, could be used by the ML system to make predictions at a higher level of abstraction than using the raw expression data. As such, the incorporation of GRN features within the ML system can improve both phenotypic prediction performance and model interpretability (Figure 3). Network topological features have found use in predicting emergent behavior in systems such as protein interaction networks and metabolic networks (Hasan et al., 2006). For example, network features have been applied to identify biologically important genes in *E. coli* metabolic networks and found their predictions to agree with genome-wide knockout screens (Plaimas et al., 2008, 2010). Similarly, ML approaches that integrate network topological features have been applied to predict metabolic pathways from correlation networks in tomato plants, identifying a novel melibiose-degradation pathway (Toubiana et al., 2019).

Designing an ML system involves many tradeoffs between detail, predictive performance, availability of data, and model interpretability. While deep learning methods provide extreme detail, incorporating GRN-derived features presents an opportunity to improve predictive performance and interpretability while still making efficient use of available data.

## Concluding Remarks and Future Perspectives

As shown in this review, multiple techniques, both empirical and *in silico* techniques, are available for the generation of GRNs. An environmental signal or a developmental cue can trigger transcriptional changes that are regulated by highly dynamic GRNs. Different transcriptomes are identified depending on the time upon stress or developmental signal (Figure 1) and as such sampling at multiple time points is crucial to fully comprehend a biological response. Moreover, as transcriptomes differ significantly between organs (root versus shoot), tissues (proliferating versus mature), and even cell types (epidermis versus stoma), the precise developmental stage at which the sampling occurs should be considered with care. Nowadays, more techniques are being developed that allow for the analysis of specific cell types using FACS, fluorescence-activated nuclei sorting (FANS), and Isolation of Nuclei TAgged in specific Cell Types (INTACT) (Bargmann and Birnbaum, 2010; Deal and Henikoff, 2011; Slane et al., 2015; Reynoso et al., 2018). Moreover, several studies report that even within the same cell type, gene expression is heterogeneous between cells. The complexity of cellular diversity and cell-to-cell gene expression variability can be addressed with transcriptomics at scale with single-cell resolution (Denyer et al., 2019). Single-cell transcriptomics allows for the simultaneous and accurate profiling of thousands of cells, revealing detailed transcriptional pathways and developmental processes (Denyer et al., 2019). Computational techniques, such as Bayesian network inference and ML approaches, will need to be adapted to the large amounts of data generated by single-cell RNA sequencing and the cross-talk between datasets.

## Author Contributions

All authors listed have made a substantial, direct and intellectual contribution to the work, and approved it for publication.

## Conflict of Interest

The authors declare that the research was conducted in the absence of any commercial or financial relationships that could be construed as a potential conflict of interest.

## Footnotes

1 http://cisbp.ccbr.utoronto.ca

2 http://neomorph.salk.edu/dev/pages/shhuang/dap_web/pages/index.php
